# Neonatal-onset multisystem inflammatory disease caused by a *de novo NLRP3* gene mutation: a case report and literature review

**DOI:** 10.3389/fped.2025.1702819

**Published:** 2025-12-19

**Authors:** Lingxia Zhao, Lingkong Zeng, Wenhao Yuan

**Affiliations:** Department of Neonatology, Wuhan Children’s Hospital (Wuhan Maternal and Child Healthcare Hospital), Tongji Medical College, Huazhong University of Science & Technology, Wuhan, Hubei, China

**Keywords:** autoinflammatory disease, canakinumab, chronic infantile neurologic cutaneous articular syndrome (CINCA), interleukin-1 inhibitors, neonatal-onset multisystem inflammatory disease (NOMID), *NLRP3* mutation

## Abstract

**Background:**

Neonatal-onset multisystem inflammatory disease (NOMID) is a rare autoinflammatory disease caused by *NLRP3* mutations, leading to excessive interleukin-1β activation and potential irreversible organ damage.

**Case description:**

We report a female neonate presenting at birth with urticaria-like rash, intermittent fever, aseptic meningitis, lymphadenopathy, and polyarthritis with persistently elevated inflammatory markers. Whole-exome sequencing revealed a heterozygous *de novo NLRP3* mutation (c.2263G>A, p.Gly755Arg), confirmed as pathogenic. Conventional therapies, including antibiotics, corticosteroids, and antihistamines, failed to achieve symptom control. Canakinumab (2–3 mg/kg per 8 weeks) was initiated, leading to rapid resolution of fever, rash, and inflammatory markers, and successful induction of clinical and biochemical remission with canakinumab during the 13-month follow-up.

**Conclusion:**

This case highlights the importance of early recognition of NOMID in neonates with antibiotic-unresponsive systemic inflammation. Early genetic confirmation and targeted IL-1 blockade with canakinumab are crucial to preventing devastating complications.

## 1 Introduction

Neonatal-onset multisystem inflammatory disease (NOMID), also known as chronic infantile neurologic, cutaneous, and articular (CINCA) syndrome, represents the most severe clinical phenotype within the spectrum of cryopyrin-associated periodic syndromes (CAPS) (1). This monogenic autoinflammatory disease is caused by gain-of-function mutations in the *NLRP3* gene, which encodes cryopyrin, a critical component of the inflammasome complex (1). These mutations lead to constitutive overproduction of interleukin-1β (IL-1β), driving uncontrolled systemic inflammation (2). Recent structural studies using cryo-EM have elucidated the mechanisms of NLRP3 inflammasome activation and inhibition, providing insights into disease pathogenesis (3).

The classic clinical triad of NOMID includes a chronic urticarial-like rash, central nervous system (CNS) involvement (typically chronic aseptic meningitis), and arthropathy, frequently accompanied by characteristic facial dysmorphism such as frontal bossing (4). The disease follows a relentlessly progressive course; without appropriate treatment, it leads to devastating complications including intellectual disability, sensorineural hearing loss, visual impairment, and secondary amyloidosis (1). Data from global registries, such as the Eurofever Registry, highlight that CNS and articular manifestations are predominant in NOMID, with significant heterogeneity in clinical presentation (5).

NOMID is exceedingly rare worldwide, with approximately 100 cases reported since its initial description (5). In China, several cases have been reported, but a comprehensive analysis of the clinical and genetic spectrum is lacking (6–21). Despite its distinctive features, diagnosis is frequently delayed by years due to its rarity and the nonspecific nature of its early symptoms, which often mimic neonatal sepsis or other inflammatory conditions (15). This diagnostic delay contributes significantly to the accumulation of irreversible organ damage. Recent studies from Japan and Europe emphasize the importance of early genetic testing to confirm *NLRP3* mutations, including somatic mosaicism, which may account for some atypical cases (22, 23).

The management of NOMID has been revolutionized by the advent of IL-1 targeted therapies. Biologic agents such as anakinra (24, 25) and canakinumab (26) have demonstrated remarkable efficacy in achieving rapid control of inflammation and halting disease progression. Long-term follow-up studies from large international cohorts, including the Eurofever Registry (27) and a nationwide Japanese survey (28) confirm that canakinumab provides sustained disease remission with a favorable safety profile. Supported by robust evidence, the 2021 EULAR/ACR points to consider endorse IL-1 inhibition as a first-line treatment for IL-1-mediated autoinflammatory diseases (29).

Here, we present a seminal case of NOMID in a Chinese neonate, representing the earliest genetically confirmed diagnosis and successful treatment initiation in China. The patient harbored a *de novo NLRP3* mutation (p.Gly755Arg) and was treated with canakinumab from 48 days of age, resulting in rapid biochemical and clinical remission. Furthermore, we provide a comprehensive systematic review and analysis of all reported Chinese NOMID cases, aiming to delineate the clinical and genetic spectrum of this disease in China and to highlight the critical importance of early recognition and intervention in the neonatal period.

## 2 Materials and methods

### 2.1 Case report

Clinical, laboratory, imaging, and genetic data were comprehensively collected from the electronic medical record system of Wuhan Children's Hospital. The diagnosis of NOMID was confirmed based on characteristic clinical features and genetic findings. Informed written consent was obtained from the parents for the publication of this case report and any accompanying images. The study was conducted in accordance with the Declaration of Helsinki and approved by the Institutional Review Board (Ethics Committee) of Wuhan Children's Hospital (Approval No: 2025R085-E01).

### 2.2 Literature review and data analysis

**Figure 1:**
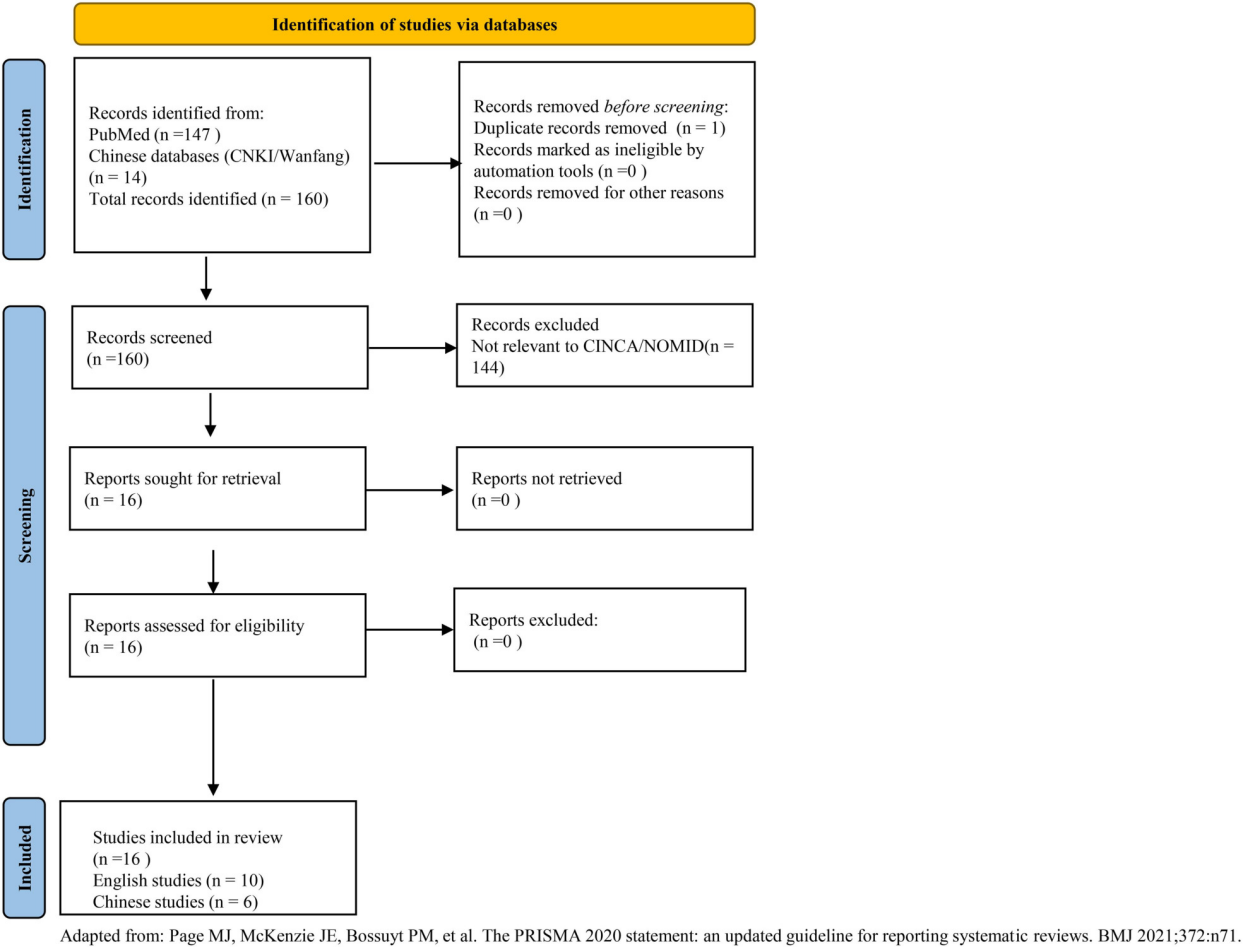
PRISMA 2020 flow diagram. Adapted from: Page et al. (30).

To comprehensively summarize the clinical characteristics of Chinese patients with NOMID, we conducted a structured literature review. The literature search was conducted up to August 2025, covering Chinese databases (China National Knowledge Infrastructure-CNKI, WanFang Data) and English databases (PubMed, Embase), with the search timeframe spanning from the inception of each database to August 2025. The search strategy employed a combination of Medical Subject Headings (MeSH) terms and keywords, including: (“NOMID” OR “CINCA” OR “Chronic Infantile Neurological Cutaneous Articular Syndrome” OR “neonatal-onset multisystem inflammatory disease” OR “NLRP3” OR “CIAS1”) AND (“China” OR “Chinese”). The search was restricted to articles published in Chinese and English, with the age restricted to the range of 0–18 years. The literature search and selection process followed the PRISMA 2020 guidelines (30). As shown in Figure 1, our search identified 161 records from PubMed and Chinese databases. After screening titles and abstracts, 144 records were excluded as irrelevant to CINCA/NOMID. The remaining 16 articles underwent full-text review, all of which met the inclusion criteria and were included in the systematic review.

All case reports and case series describing patients of Chinese ethnicity with a clinical and/or genetic diagnosis of NOMID/CINCA were included. Data extraction was performed using a standardized form. The extracted information included: (1) demographic data (gender, current age, age of onset, age at diagnosis); (2) clinical manifestations (rash, fever, CNS involvement, musculoskeletal symptoms, organomegaly, etc.); (3) laboratory and imaging findings; (4) genetic results. Data were summarized descriptively and are presented in Supplementary Table S1.

### 2.3 Risk of bias assessment

Given that all studies included in this systematic review are case reports or small case series, a formal risk of bias assessment using tools designed for controlled trials (e.g., the Cochrane Risk of Bias tool) was not applicable. The inherent limitations of case reports, including their retrospective nature, potential for selection bias, and inconsistent reporting of outcomes, are acknowledged. To ensure data quality and reliability, we evaluated each included case based on the completeness of reporting across four key domains: (1) patient demographics, (2) clinical manifestations, (3) genetic confirmation of the NLRP3 mutation, and (4) treatment regimen and response. Cases with critically missing information in these domains were excluded during the screening phase.

## 3 Case presentation

A female infant was born at 37^+6^ weeks gestation via spontaneous vaginal delivery through grade III meconium-stained amniotic fluid. The pregnancy was otherwise uncomplicated. Birth weight was 2,700 g (25th percentile). The parents were non-consanguineous and had no significant family history.

**Figure 2:**
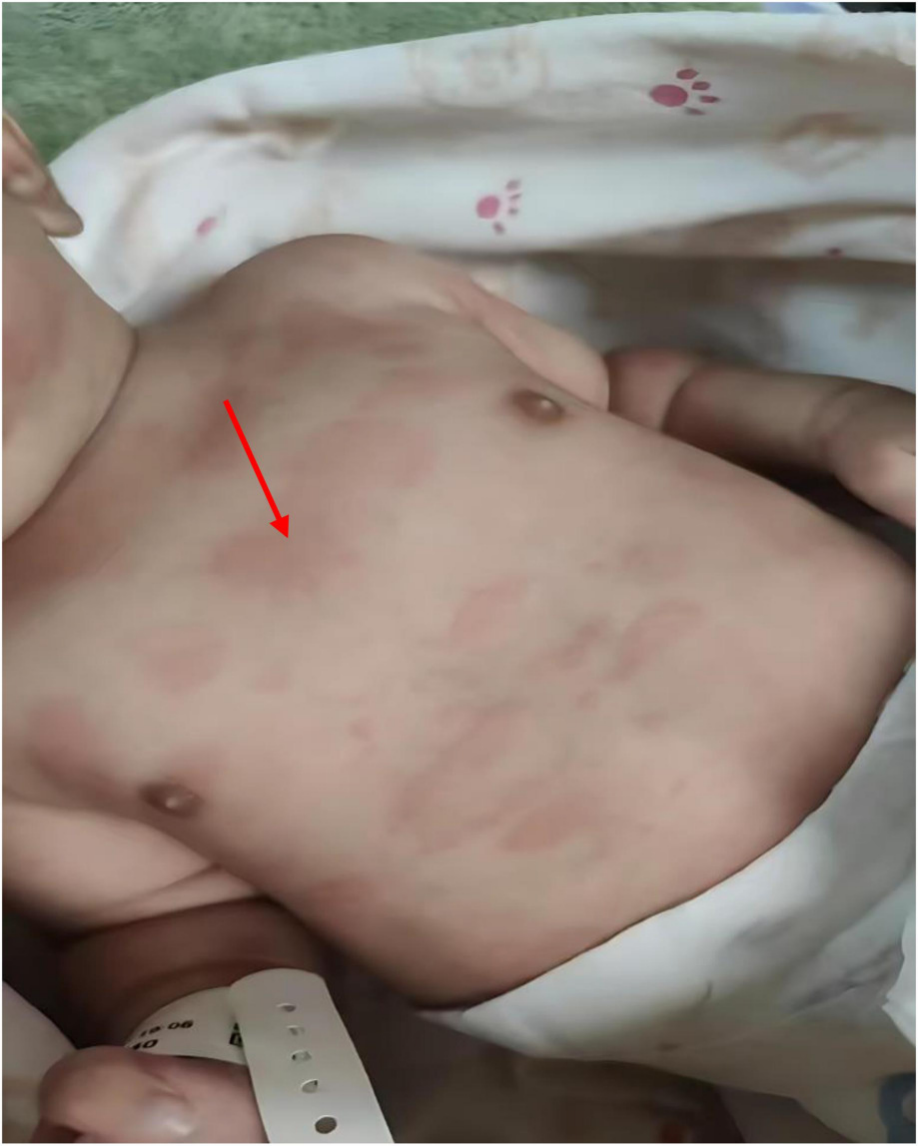
Urticarial-like rash observed at birth: scattered erythematous plaques of variable size, some coalescing into larger patches. Rash was blanchable on pressure and persisted despite empirical treatment. (The arrow points to the rash).

A generalized urticarial-like rash was noted on the first day of life (Figure 2). The infant was admitted to a local neonatal intensive care unit (NICU) due to concerns of early-onset sepsis, particularly given the history of meconium-stained liquor. Initial laboratory investigations revealed significantly elevated inflammatory markers: white blood cell count (WBC) 29.95 × 10⁹/L (reference: 5–21) and C-reactive protein (CRP) 35.06 mg/L (reference: < 3). Despite administration of broad-spectrum antibiotics (Ampicillin 50 mg/kg per dose, q12 h, and Meropenem 20 mg/kg per dose, q12 h, for 7 days) and intravenous methylprednisolone (2 mg/kg/day, for 2 days), the rash persisted, and inflammatory markers remained elevated.

Due to the persistent, unexplained inflammation, the infant was transferred to our tertiary NICU on the 8th day of life for further management. On admission, physical examination was significant for frontal bossing, a widespread, migratory, blanching urticarial rash with areas of confluence, and palpable axillary lymphadenopathy (approximately 1 cm in diameter). Examination of the lower limbs revealed a symmetrical arthropathy involving the knees and ankles, characterized by swelling, and tenderness on passive movement, resulting in reduced spontaneous activity. Recurrent febrile episodes (temperature 38–38.3°C) occurred every 2–6 days, each lasting less than 24 h. The clinical course over the subsequent weeks was characterized by recurrent febrile episodes and fluctuating rash intensity, despite multiple empirical therapies.

A comprehensive diagnostic workup was pursued to elucidate the cause of the refractory inflammation. Lumbar puncture performed on day 9 revealed CSF findings indicative of aseptic meningitis: pleocytosis (105 × 10^6^/L), elevated protein (1.32 g/L), and hypoglycorrhachia (1.68 mmol/L). Cranial MRI on day 14 was unremarkable. Initial automated auditory brainstem response (AABR) showed a “refer” result in the left ear. Critically, extensive microbiological investigations returned negative.

The patient's hospital course was characterized by persistent symptoms despite multiple empirical therapeutic interventions. She was initially treated with a combination of intravenous Ampicillin and Meropenem, which was later adjusted to vancomycin in combination with Meropenem. Additional interventions included antifungal therapy (Fluconazole), intravenous immunoglobulin (IVIG), and a trial of intravenous Methylprednisolone, which provided only transient and incomplete symptom control (see Supplementary Table S2 for detailed timeline).

Given the constellation of findings—early-onset urticarial rash, chronic meningitis, arthropathy, frontal bossing, and persistently elevated inflammatory markers unresponsive to conventional therapies—a strong suspicion for a monogenic autoinflammatory disease, was raised. After obtaining informed consent from the parents, whole-exome sequencing (WES) was performed.

**Figure 3:**
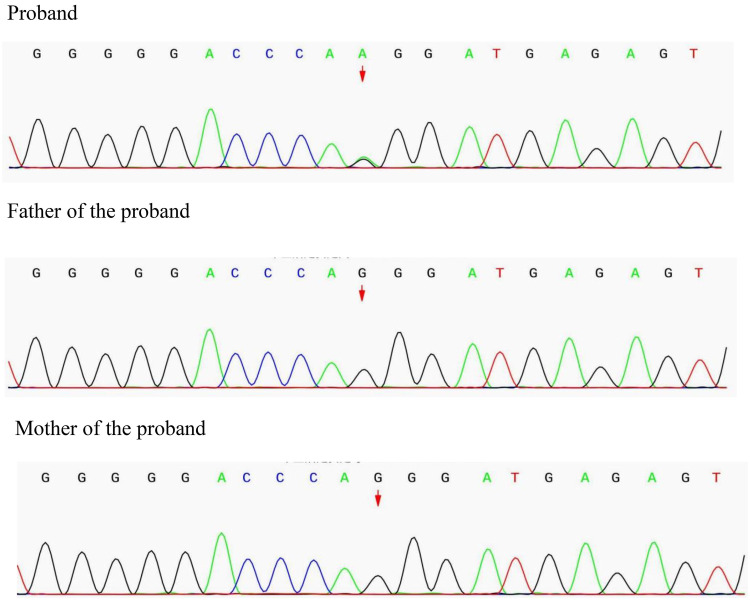
Genetic findings in the proband and parents: Sanger sequencing confirmed a heterozygous missense mutation in the NLRP3 gene (c.2263G>A, p.Gly755Arg) in the proband. Both parents carried the wild-type all ele, confirming the mutation as *de novo*.

Genetic analysis revealed a heterozygous missense variant (c.2263G>A) in exon 4 of the *NLRP3* gene, resulting in an amino acid change from glycine to arginine at position 755 (p.Gly755Arg). Sanger sequencing confirmed the presence of this variant in the proband and its absence in both parents, confirming its *de novo* origin (Figure 3). This variant was interpreted as pathogenic according to the American College of Medical Genetics and Genomics (ACMG) standards and guidelines (evidence codes: PS2, PS3_MOD, PS4_MOD, PM2_SUP, PP2, PP3), confirming the diagnosis of NOMID.

Targeted therapy with subcutaneous canakinumab was initiated at 48 days of life (2 mg/kg every 8 weeks). The response was rapid, with resolution of rash and fever within 24 h and normalization of inflammatory markers. A transient CRP rise to 10 mg/L before the second dose led to a dose increase to 3 mg/kg every 8 weeks. Thereafter, the patient achieved clinical and serological remission during 13 months of follow-up (Supplementary Table S2).

**Figure 4:**
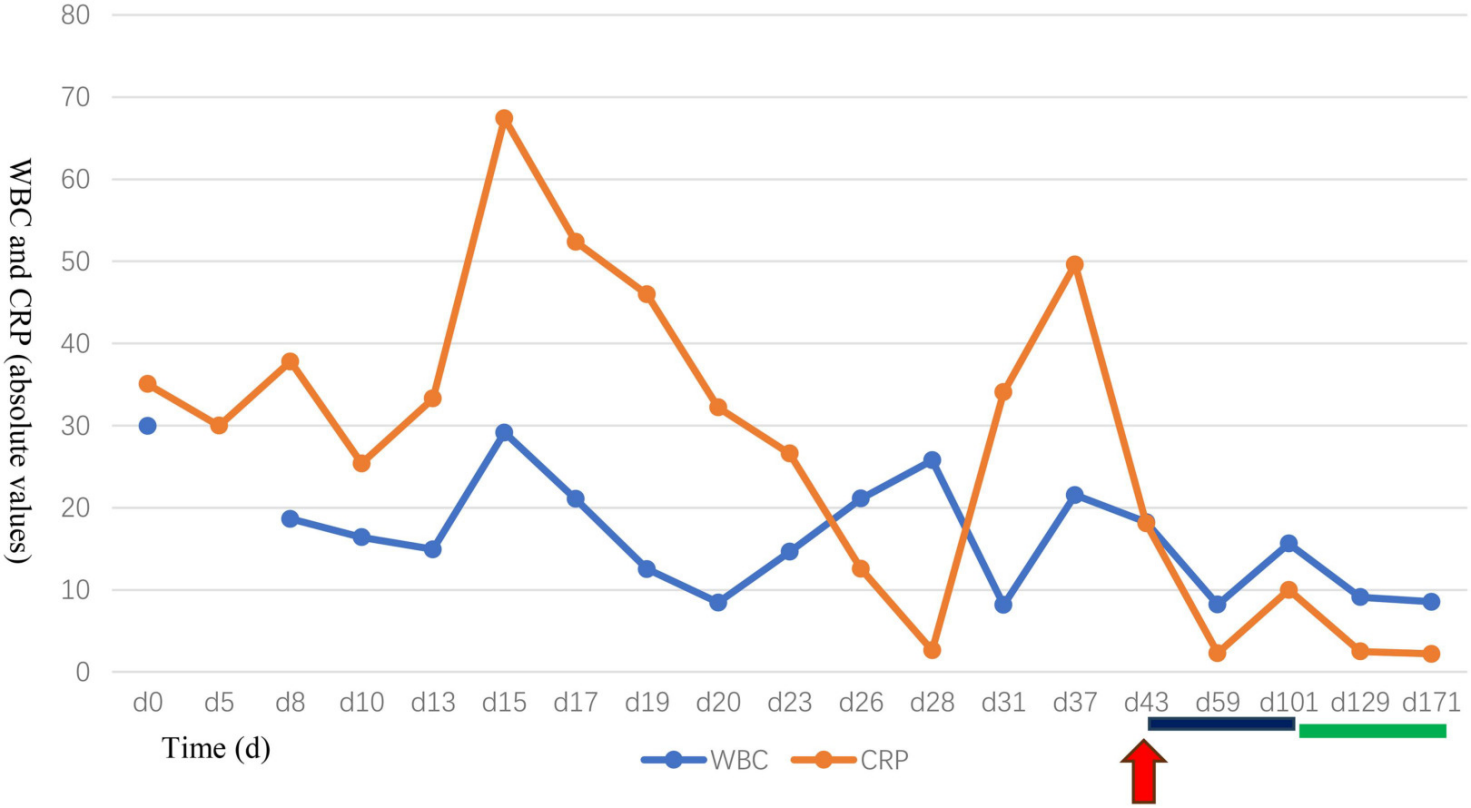
Longitudinal changes in white blood cell count and C-reactive protein following canakinumab treatment in a case of neonatal-onset multisystem inflammatory disease. The red arrow indicates the time of definitive diagnosis and initiation of canakinumab treatment. The black annotation denotes the canakinumab dosage of 2 mg/(kg·day). During follow-up, a mild elevation in both white blood cell (WBC) count and C-reactive protein (CRP) was observed. After the dosage was adjusted to 3 mg/kg/day, both WBC and CRP decreased to within the normal range.

Monitoring and Outcomes: A standardized protocol was implemented to monitor efficacy, adverse events, and infections. This included:

a. Bi-monthly assessments: Complete blood count, CRP, liver and renal function, BAEP, and fundoscopic examinations (Figure 4).
b. Quarterly assessments: Neurodevelopmental evaluation using the Gesell Developmental Scales (Supplementary Table S3).
c. All monitored parameters remained normal throughout follow-up, confirming sustained remission without treatment-related complications or disease recurrence.
d. Growth: Despite clinical remission, her linear growth and weight gain remained delayed, with both parameters persistently below the 10th percentile.

## 4 Discussion

We present a seminal case of NOMID in a Chinese neonate, notable for several key aspects: it is the earliest genetically confirmed case in China, it harbors a rare *NLRP3* (p.Gly755Arg) mutation, and it demonstrates the profound efficacy of very early intervention with canakinumab, initiated at 48 days of life, in achieving clinical and biochemical remission without emerging serious complications during the 13-month follow-up.

**Figure 5:**
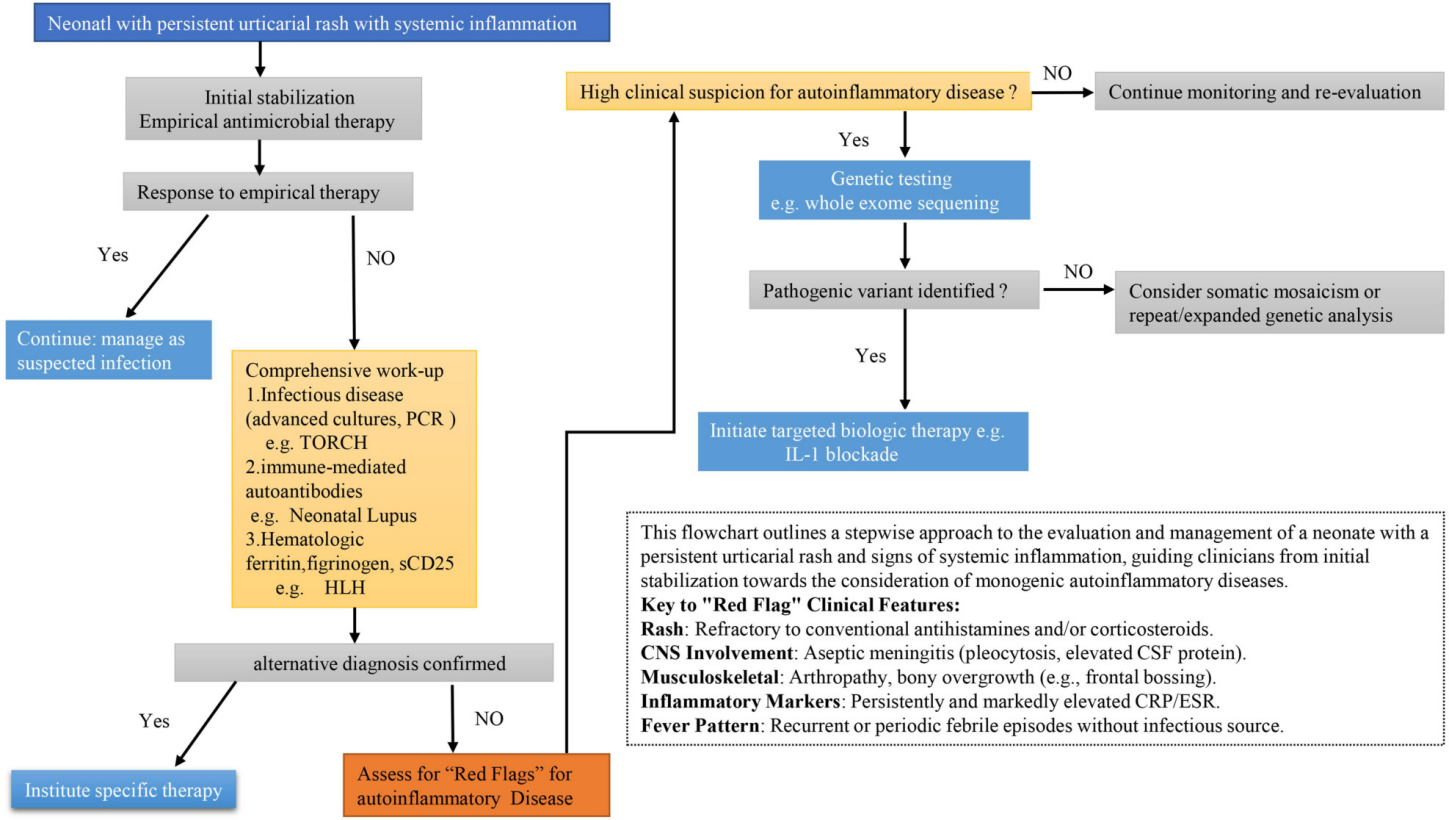
Diagnostic and management pathway for neonates with persistent Urticaria and systemic inflammation.

The clinical presentation of our patient is a textbook example of the classic NOMID triad, manifesting with urticarial rash, aseptic meningitis, and joint involvement within the first week of life. The initial presentation of our patient—characterized by neonatal-onset fever, urticarial rash, and elevated inflammatory markers—understandably prompted a broad differential diagnosis. It was crucial to systematically rule out more common entities before arriving at the rare diagnosis of NOMID. First, severe bacterial infections, including neonatal sepsis and meningitis, were primary concerns. However, the persistence of symptoms despite broad-spectrum antibiotic coverage, coupled with consistently negative bacterial cultures (blood and CSF) and normal procalcitonin levels, argued strongly against an ongoing bacterial process. Second, congenital infections (e.g., TORCH syndrome) were considered due to the multi-system involvement. These were effectively excluded by negative serological and PCR testing for relevant pathogens. Third, the urticarial rash and systemic inflammation led to the consideration of neonatal lupus erythematosus. This was ruled out by the absence of maternal anti-Ro/SSA and anti-La/SSB antibodies. Finally, the fulminant systemic inflammatory response and highly elevated ferritin raised the possibility of hemophagocytic lymphohistiocytosis (HLH). Nevertheless, key diagnostic criteria for HLH, such as persistent cytopenias, significant hypofibrinogenemia, and markedly elevated soluble CD25 levels, were absent. The constellation of sterile meningitis, characteristic bony involvement (frontal bossing), and a steroid-refractory, relapsing-remitting course ultimately pointed towards a monogenic autoinflammatory disease, guiding the decision to pursue genetic testing which confirmed NOMID. The clinical management pathway for neonates with persistent urticaria and systemic inflammation is summarized in the flowchart in Figure 5.

Our systematic review of 52 Chinese NOMID cases (6–21) provides the most comprehensive characterization of this disease in the Chinese population to date. The clinical manifestations were largely consistent with those reported in other international cohorts (5, 28) (Supplementary Table S4), with fever (96.6%) and skin rash (100%) being the most common features, followed by frequent central nervous system involvement (86.6%) and musculoskeletal abnormalities (82.1%). However, the prevalences of hearing impairment, ocular involvement, and renal amyloidosis in our cohort were lower than those in the Japanese cohort. This discrepancy may be attributed to the retrospective nature of our study, which is susceptible to under-reporting bias, combined with the relatively short follow-up duration, potentially leading to an underestimation of long-term sequelae accumulation. In contrast, when compared to the mixed international CAPS cohort, both the Chinese and Japanese NOMID cohorts exhibited a higher overall burden of complications, further validating NOMID as the most severe phenotypic subtype within the CAPS spectrum.

Our patient exhibited significant growth delay (length and weight below the 10th percentile) at the time of diagnosis. This is a well-documented feature in infants with NOMID and is multifactorial in origin (1, 5). The primary driver is believed to be the chronic systemic inflammation itself. The persistent overproduction of IL-1β and other pro-inflammatory cytokines creates a catabolic state, disrupts the growth hormone axis, and suppresses appetite, leading to inadequate nutritional intake (1, 15). Furthermore, joint involvement and arthropathy can contribute to discomfort and functional limitations, potentially exacerbating feeding difficulties and reducing physical activity, which may indirectly impact growth (5). While the contribution of chronic inflammation and nutritional status is paramount, a direct inhibitory effect of cytokines on chondrocyte function and bone growth plates is also postulated (1). The observed catch-up growth following the initiation of canakinumab, which led to rapid inflammatory control, strongly supports the central role of IL-1β-driven inflammation as the underlying cause rather than the treatment itself. This aligns with long-term follow-up studies of canakinumab-treated CAPS patients, which have not identified a significant adverse effect on growth; conversely, therapy often allows for the amelioration of pre-existing growth failure (27, 28).

The p.Gly755Arg variant identified in our patient is located within the leucine-rich repeat (LRR) domain of the NLRP3 protein. This domain is involved in protein-protein interactions and autoinhibition. Mutations in this region are mechanistically intriguing; recent evidence suggests they can promote NLRP3 hyperactivation through multiple mechanisms, including destabilizing autoinhibition, enhancing NEK7-driven inflammasome assembly, and impairing regulatory ubiquitination, leading to protein accumulation (31). Recent high-resolution cryo-EM structural studies—including the visualization of the active NLRP3 inflammasome disc, the inhibited NLRP3 decamer, and the full-length NLRP3 “cage”—have definitively established that the LRR domain plays a central role in regulating NLRP3 activation by maintaining an autoinhibited conformation (3, 32, 33). These structural models posit that pathogenic mutations within the LRR domain, such as p.Gly755Arg, likely lead to the release of this autoinhibited state by disrupting critical intramolecular interfaces, thereby resulting in spontaneous NLRP3 activation (3, 32, 33). This mechanism, grounded in structural biological findings, provides a powerful explanation for the severe, neonatal-onset NOMID phenotype observed in our case. Clinically, variants in the LRR domain have been associated with a severe phenotype and a reportedly higher incidence of sensorineural hearing loss (31). The fact that our patient's hearing normalized completely after treatment is therefore a powerful testament to the remarkable efficacy of preemptive IL-1 blockade in preventing this devastating complication. To date, only five other cases with this specific variant have been reported globally (4, 22, 23, 34, 35) all of whom presented with the severe classic NOMID phenotype, reinforcing its pathogenicity.

It is crucial to note that approximately one-third of patients with a classic clinical phenotype of NOMID do not have an identifiable germline mutation on standard Sanger sequencing (22, 23). This is often due to somatic NLRP3 mosaicism, which requires next-generation sequencing techniques on DNA from affected tissues (e.g., skin, blood subpopulations) or highly sensitive blood tests for detection. Therefore, a negative genetic test should not definitively rule out the diagnosis in a highly suggestive clinical context, and further specialized testing should be pursued.

The management of NOMID/CINCA syndrome has been revolutionized by IL-1 inhibitors, including anakinra and canakinumab (1). For the most severe phenotype of NOMID, potent IL-1 blockade with either anakinra or canakinumab is required (1, 29).

Anakinra demonstrates rapid efficacy, with landmark studies confirming its ability to control systemic inflammation and ameliorate neurological manifestations (24). Its sustained effectiveness and safety profile have been further established through long-term follow-up studies (25). However, its requirement for daily subcutaneous injections (1–2 mg/kg/day) can present challenges for long-term adherence, particularly in pediatric patients (1, 24, 25).

In contrast, canakinumab offers superior dosing convenience due to its prolonged half-life, allowing for subcutaneous administration every 8 weeks (2–4 mg/kg, with a maximum of 150 mg for children weighing 7–40 kg) (26). Large-scale, real-world studies and registry data have consistently validated its exceptional efficacy in achieving and maintaining complete disease remission, while significantly improving treatment adherence and quality of life (27, 28).

For our case, canakinumab was selected based on drug availability and long-term adherence considerations. Treatment achieved remarkable outcomes: rapid normalization of inflammatory markers, complete rash resolution, normalized CSF cell counts, and significant hearing improvement. The choice of canakinumab was also supported by emerging evidence of its favorable safety profile and dosing convenience in very young infants. This case of early-onset NOMID confirms canakinumab's excellent efficacy and safety in very young patients, providing valuable guidance for clinical decision-making in similar scenarios.

However, it is important to note that the follow-up period of 13 months, while demonstrating excellent initial response, is relatively short. Long-term complications of NOMID, including hearing loss, visual impairment, and amyloidosis, may develop later in life. Therefore, continuous long-term monitoring of our patient is essential to fully assess the sustained efficacy and safety of canakinumab therapy.

The objective longitudinal data presented in our case underscore several key points. First, the rapid normalization of CRP and WBC following canakinumab initiation (Figure 4) provides serological confirmation of the drug's efficacy in suppressing NLRP3-mediated inflammation. Second, the normalization of BAEP findings and the absence of ocular pathology demonstrate the critical role of early IL-1β blockade in preventing irreversible organ damage, particularly to the nervous system. Finally, the formal developmental assessment confirming age-appropriate milestones strengthens the evidence that controlling neuroinflammation from early infancy can preserve neurodevelopmental potential.

Diagnosing NOMID in the neonatal period remains challenging due to the absence of formal, validated criteria for this age group. In the present case, the diagnosis was established by integrating proposed clinical features from established classification systems such as the Eurofever/PRINTO criteria (36), the presence of the classic triad (urticarial rash, aseptic meningitis, and arthropathy), rigorous exclusion of alternative diagnoses, and definitive genetic confirmation of a pathogenic NLRP3 mutation (37). This case report, along with other real-world evidence (38), underscores the urgent need to develop more sensitive and standardized diagnostic algorithms for early infancy, as prompt recognition is critical to prevent life-long sequelae.

## 5 Study limitations

This study has several limitations that should be considered. First, its retrospective design, reliant on previously published case reports, may be subject to publication and selection biases. Second, the 13-month follow-up period, while demonstrating excellent initial outcomes, is relatively short for a chronic lifelong disease like NOMID. The risk of long-term sequelae, such as hearing loss, amyloidosis, or visual impairment, persists. Consequently, continuous, long-term monitoring of organ development, neurocognitive function, and potential adverse effects of biologic therapy is essential to fully ascertain the sustained efficacy and safety of canakinumab in this patient.

## 6 Conclusion

This case and the accompanying literature review deliver several critical messages for clinicians, particularly neonatologists and pediatric intensivists:

1. NOMID can present on the first day of life. A persistent urticarial rash in a neonate should never be dismissed and should immediately prompt consideration of autoinflammatory etologies.
2. The diagnostic delay in China is unacceptably long. Closing this gap requires increased awareness and a lower threshold for genetic testing in neonates with unexplained inflammation.
3. Genotype-Phenotype Correlations exist but should not deter testing. Even rare variants in domains like LRR can cause severe disease.
4. Early treatment is paramount. The excellent outcome in our patient, compared to the significant morbidity in historically delayed cases, provides compelling evidence that prompt genetic diagnosis and immediate initiation of IL-1 blockade can fundamentally alter the natural history of NOMID, preventing lifelong disability and ensuring normal development. Canakinumab is effective and safe even in early infancy.
5. Somatic Mosaicism is a key consideration in mutation-negative patients with a classic phenotype.

In conclusion, this report underscores NOMID as a devastating but treatable cause of neonatal systemic inflammation. Vigilance, early genetic testing, and swift targeted therapy are the keys to transforming outcomes for these children.

## Data Availability

The datasets presented in this study can be found in online repositories. The names of the repository/repositories and accession number(s) can be found below: https://www.ncbi.nlm.nih.gov/snp/, rs1305028279.

## Supplementary material

Supplementary Table 1 Clinical characteristics of 52 neonatal cases with NOMID reported in China.

Supplementary Table 2 Timeline of Clinical Presentation, Diagnostic Workup, Treatment, and Response.

Supplementary Table 3 Developmental Assessment Using the Gesell Developmental Schedules During Follow-up.

Supplementary Table 4 Comparative Analysis of Demographic, and Clinical Features among Chinese NOMID Cohort, International CAPS Cohort, and Japanese NOMID Cohort.
