# Correction: Neonatal-onset multisystem inflammatory disease caused by a *de novo* *NLRP3* gene mutation: a case report and literature review

**DOI:** 10.3389/fped.2026.1787121

**Published:** 2026-02-04

**Authors:** Frontiers Production Office

**Affiliations:** Frontiers Media SA, Lausanne, Switzerland

**Keywords:** autoinflammatory disease, canakinumab, chronic infantile neurologic cutaneous articular syndrome (CINCA), interleukin-1 inhibitors, neonatal-onset multisystem inflammatory disease (NOMID), *NLRP3* mutation

Article type

The article type was erroneously given as Original Research. The correct article type is Case Report.

The original version of this article has been updated.

