# Supplementary material for: Neonatal-onset multisystem inflammatory disease caused by a de novo NLRP3 gene mutation: a case report and literature review
Source: Front Pediatr. 2025 Dec 19;13:1702819. doi: 10.3389/fped.2025.1702819 (PMC12757356; doi:10.3389/fped.2025.1702819)
Supplement: Supplementary Table 1 — Clinical characteristics of 52 neonatal cases with NOMID reported in China. [file Table1.docx]

**Supplementary Table 1** Clinical characteristics of 52 neonatal cases with NOMID reported in China.

| **Author, Year (Ref)** | Guan  et al.  2014  (6) | Fu  et al.  2014  (7) | Zhao  et al.  2015  (8) | Zhang  et al.  2019  (9) | Luo  et al.  2020  (10) | Jiang  et al.  2021  (11) | Zhou  et al.  2022  (12) | An  et al.  2023  (13) |
| --- | --- | --- | --- | --- | --- | --- | --- | --- |
| **No.** | 1 | 2 | 3 | 4-13 | 14,15 | 16 | 17-24 | 25 |
| **Gene** | c.1702T>A  p.F568I | c.907G>A  p.D303N | c.2667G>T  p.K889N | c.913G>A  p.D305N  c.1057G>T  p.V353L  c.1702T>A  p.F568I)  c.1703T>A  p.F568Y)  c.1710G>C  p.K570N  c.1789A>G  p.S597G  c.1991T>C  p.M664T  c.2269G>A  p.G757R | c.1568T>A  p.F523Y  c.1330T>G  p.F444V | c.913G>A  p.D305N | c.1715A>G  p.Y572C  c.1711G>C  p.G571R  c.1991T>C  p.M664T  c.1991T>C  p.M664T  c.983G>A  p.G328E  c.913G>A  p.D305N  c.918G>T  p.E306D  c.1082T>G  p.L361W | c.1309A>G  p.K437E |
| **Age at Diagnosis** | 1 year | 20 years | 7.7 years | 7 mo- 16 years (6.4years) | 8 mo  3 mo | 3.5 years | ND | 1 mo |
| **Sex** | M | F | M | 3M | F  M | M | ND | M |
| **Age of Onset** | 2 d | After birth | 8 mo | 3 h- 6 years | 2 d  1 d | After birth | ND | 1h |
| **Fever** | + | + | + | 10/10 | +  + | + | 7/8 | + |
| **Rash** | + | + | + | 10/10 | +  + | + | 8/8 | + |
| **CNS Involvement** | + | + | - | 9/10 | +  + | - | 8/8 | + |
| **MSK Involvement** | + | ND | - | 9/10 | ND | + | 6/8 | + |
| **Hearing Loss** | + | + | + | 8/10 | ND | - | 4/8 | - |
| **Eye Involvement** | + | ND | + | 7/10 | +  + | + | 3/8 | + |
| **Cognitive Impairment** | ND | - | ND | 8/10 | ND | ND | ND | + |
| **Impaired Growth** | + | ND | ND | 10/10 | ND | ND | 3/8 | + |
| **Abnormal facies** | + | + | - | 10/10 | +  ND | ND | ND | + |
| **Lymphadenopathy/ Hepatosplenomegaly** | + | + | - | 6/10 | ND | ND | ND | + |

**Supplementary Table 1** Continued

| **Author, Year (Ref)** | Xie  et al.  2023  (14) | Zhao  et al  2024  (15) | Shangguan  et al  2024  (16) | Zhang  et al.  2023  (17) | Shu  et al.  2023  (18) | Zhu  et al.  2024  (19) | Ma  et al.  2024  (20) | Ruan  et al.  2025  (21) | Our case |
| --- | --- | --- | --- | --- | --- | --- | --- | --- | --- |
| **No.** | 26 | 27 | 28,29 | 30,31 | 32-43 | 44-49 | 50 | 51 | 52 |
| **Gene** | c.1718T>G，p.L573W | c.1210G>C  p.V404L | c.1311G>T  p.K437N  c.785G >C  p.R262P | ND  ND | c.1336T> G  p. F446V  c.1223T > C  p. M408T  c.1710G > T  p. K570N  c.931T > G  p. F311V  c.796C>T  p. L266F  c.1223T > C  p. M408T  c.1703T>A  p. F568Y  c.796C>T   1. L266F   c. 907G>T  p. D303N  c. 998G>T  p.S333I  c.913G>A  p. D305N  c.1711G>C  p. G571R | c.797T>C  p.L266P  c.1006A>G  p.I336V  c.1710G > T  p.K570N  c.1892G>T  p.C631F  c.1444A>T  p.I482F  c.1210G>C  p.V404L | c.1444A>T  p.I482F | c.1006A>G  p.I336V | c.2263G>A  p.G755R |
| **Age at Diagnosis** | 2.9 years | 1 mo | 13.5 years  14 mo | ND | 1.5years | 9.5mo | 10years | 4mo | 1 mo |
| **Sex** | F | F | M  F | M  F | 5F  7M | 3M  3F | M | F | F |
| **Age of Onset** | After  birth | 12h | neonate | ND | 1-4d | 2.5d | 6mo | neonate | 1h |
| **Fever** | + | + | 2/2 | 1/2 | 12/12 | 6/6 | + | + | + |
| **Rash** | + | + | 2/2 | 2/2 | 12/12 | 6/6 | + | + | + |
| **CNS Involvement** | - | + | 2/2 | 2/2 | 11/12 | 6/6 | + | + | + |
| **MSK Involvement** | + | - | 2/2 | -  - | 10/12 | 5/6 | + | - | + |
| **Hearing Loss** | - | - | ND | 2/2 | 6/12 | 4/6 | + | + | + |
| **Eye Involvement** | - | - | +  ND | 2/2 | 8/12 | 5/6 | ND | - | - |
| **Cognitive Impairment** | ND | - | ND | 2/2 | - | - | + | ND | - |
| **Impaired Growth** | + | - | ND  ND | 2/2 | 8/12 | 4/6 | + | ND | + |
| **Abnormal facies** | + | - | ND  + | ND | 12/12 | 1/6 | + | ND | + |
| **Lymphadenopathy/ Hepatosplenomegaly** | - | - | +  ND | ND | 3/12 | 2/6 | + | ND | + |

Legend: This table summarizes the demographic, clinical, and genetic features of 29 included Chinese NOMID patients. Abbreviations: M, male; F, female; d, day(s); mo, month(s); h, hour(s); CNS, central nervous system; MSK, musculoskeletal; ND, no data or not described in the original report. Symbols: "+", present; "-", absent. For studies reporting multiple patients (e.g., Zhang et al., 2019), data are presented as the number of affected patients over the total number of patients in the cohort (e.g., 10/10).F,famale.M,male.
