# Supplementary material for: Neonatal-onset multisystem inflammatory disease caused by a de novo NLRP3 gene mutation: a case report and literature review
Source: Front Pediatr. 2025 Dec 19;13:1702819. doi: 10.3389/fped.2025.1702819 (PMC12757356; doi:10.3389/fped.2025.1702819)
Supplement: Supplementary Table 2 — Timeline of Clinical Presentation, Diagnostic Workup, Treatment, and Response. [file Table2.docx]

Supplementary Table 2: Timeline of Clinical Presentation, Diagnostic Workup, Treatment, and Response

| Time (Day of Life) | Key Clinical Signs & Symptoms | Key Laboratory & Imaging Findings | Treatment Interventions | Treatment Response& Outcomes |
| --- | --- | --- | --- | --- |
| D0-7 | urticarial rash | WBC↑ (29.95x10⁹/L),  CRP↑ (35.06 mg/L) | Ampicillin(50mg/kg,q12h,for7 days),  Meropenem(20mg/kg,q12h,for7days)  Methylprednisolone(2mg/kg/d,for2days) | No response |
| D8(Transferred) | urticarial rash | WBC↑(18.41x10⁹/L),  CRP↑ (34.60mg/L) | Ampicillin(50mg/kg,q12h,for2days),  Meropenem(20mg/kg,q8h,for1day) | - |
| D9 | urticarial rash | CSF Abnormal: Cells↑(23), Protein↑(1.32), Glucose↓(1.68);  BAEP: Left ear fail | Meropenem(40mg/kg,q8h,for12days)  Cetirizine | Disease activity ongoing |
| D12 | urticarial rash | CSF:Marked pleocytosis↑(105),  Protein↑(1.35),  Glucose↓(2.05);  CSF NGS: S. epidermidis/C. parapsilosis | Vancomycin(15mg/kg,q8h,for18d), Fluconazole(6mg/kg,qd,for14d) added | Inflammation uncontrolled |
| D14 | Fever (38.3°C)  urticarial rash | Brain MRI: No significant abnormalities | Dexamethasone(0.5mg/kg/d,for1day), IVIG(2.5g,for2days) | Transient improvement |
| D15 | Rash resolved completely | - | - | Steroids/IVIG effective but transient |
| D16-17 | Fever recurrence (38°C, 37.7°C) | CRP peak (67.40mg/L) | - | - |
| D23 | Fever (38°C), increased rash | CRP↑ (25.60mg/L) | Chlorpheniramine  Methylprednisolone(2mg/kg/d,for5days)  IVIG(5g,for2days) | - |
| D26-28 | Rash improved | CRP(2.63mg/L) | - | Steroid-dependent, poor control |
| D29 | Fever (38.3°C) & rash recurrence | - | - | - |
| D31 | Fever (38.2°C), rash | CRP elevated again (34.06mg/L);  ANA 1:100 | Meropenem(20mg/kg,q8h,for14days),  Methylprednisolone restarted(2mg/kg/d,for5days),  reduce(1mg/kg/d,for5days) | Persistent symptoms,  abnormal labs |
| D45 | rash | Genetic Dx: NLRP3 p.Gly755Arg | - | - |
| D48 | rash | - | All antibiotics/steroids stopped;  First Canakinumab (2 mg/kg) | Turning Point |
| After 1 day of treatment with Canakinumab | Afebrile, rash resolved | - | - | Rapid clinical response |
| After 2 weeks of treatment with Canakinumab | No fever/rash | WBC(8.21x10⁹/L),  CRP (2.3mg/L) | - | Labs response |
| After 8 weeks of treatment with Canakinumab | Clinically well | Transient elevation  WBC(15.63x10⁹/L),  CRP (10mg/L) | Canakinumab dose increased to 3 mg/kg | CRP normalized after adjustment |
| After 12 weeks of treatment with Canakinumab |  | WBC(9.12x10⁹/L),  CRP (2.5mg/L) |  |  |
| After 16 weeks of treatment with Canakinumab |  | WBC(8.55x10⁹/L),  CRP (2.2mg/L) |  |  |
| 13-month | catch-up growth | Normal serial labs; Normal BAEP; Normal Gesell | Canakinumab (3 mg/kg/q8w) | clinical & biochemical remission |

Table footnote:

BAEP, brainstem auditory evoked potential; WBC, white blood cell; CRP, C-reactive protein; ANA, antinuclear antibody; IVIG, intravenous immunoglobulin.
