# Supplementary material for: Neonatal-onset multisystem inflammatory disease caused by a de novo NLRP3 gene mutation: a case report and literature review
Source: Front Pediatr. 2025 Dec 19;13:1702819. doi: 10.3389/fped.2025.1702819 (PMC12757356; doi:10.3389/fped.2025.1702819)
Supplement: Supplementary Table 3 — Developmental Assessment Using the Gesell Developmental Schedules During Follow-up. [file Table3.docx]

**Supplementary Table 3. Developmental Assessment Using the Gesell Developmental Schedules During Follow-up**

| Domain | 6 monts | 9 months | 12 months |
| --- | --- | --- | --- |
| Adaptive behavior | 103 | 102 | 105 |
| Gross motor | 105 | 104 | 102 |
| Fine motor | 101 | 103 | 106 |
| language | 98 | 100 | 102 |
| Personal-social behavior | 102 | 104 | 105 |
| DQ | 102 | 103 | 104 |
