# Supplementary material for: Neonatal-onset multisystem inflammatory disease caused by a de novo NLRP3 gene mutation: a case report and literature review
Source: Front Pediatr. 2025 Dec 19;13:1702819. doi: 10.3389/fped.2025.1702819 (PMC12757356; doi:10.3389/fped.2025.1702819)
Supplement: Supplementary Table 4 — Comparative Analysis of Demographic, and Clinical Features among Chinese NOMID Cohort, International CAPS Cohort, and Japanese NOMID Cohort. [file Table4.docx]

| **Parameter**  **(Ref)** | **Chinese(n=30)**  **( This Study )** | **International(n=136)**  **(5)** | **Japanese(n=21)**  **(23)** |
| --- | --- | --- | --- |
| **Demographics** |  |  |  |
| ‧ Median Age at Diagnosis(year) | **1.3** (0.1 - 20 ) | 15(5-36) | 1(0.1-64.5) |
| ‧ Male Sex, n (%) | 55.2% | 50% | 71.4% |
| **Clinical Manifestations, n (%)** |  |  |  |
| ‧ Urticarial Rash | 100%(30/30) | 97% | 100% |
| ‧ Fever | 96.6%(29/30) | 84% | 90.5% |
| ‧ Articular/Musculoskeletal Involvement | 82.1%(23/28) | 86% | 90.5% |
| ‧ CNS Involvement | 86.6%(26/30) | 40% | 95.2% |
| ‧ Sensorineural Hearing Loss | 66.6%(18/27) | 42% | 76.2% |
| ‧ Eye Involvement | 60.7%(17/28) | 71% | 76.2% |
| ‧ Impaired Growth | 70.8%(17/24) | 70% | 61.9% |
| AA amyloidosis | 0 | 4% | 14.3% |

**Supplementary Table 4.** Comparative Analysis of Demographic, and Clinical Features among Chinese NOMID Cohort, International CAPS Cohort, and Japanese NOMID Cohort

**Notes:** The Chinese cohort and Japanese cohort consist exclusively of patients with NOMID, while the International cohort includes all CAPS patients from the Eurofever Registry.

CAPS, cryopyrin-associated periodic syndrome. NOMID, neonatal-onset multisystem inflammatory disease; CNS, central nervous system. MID Cohort, Japanese NOMID Cohort and International CAPS Cohort.
